# Cell-length heterogeneity: a population-level solution to growth/virulence trade-offs in the plant pathogen *Dickeya dadantii*

**DOI:** 10.1371/journal.ppat.1007703

**Published:** 2019-08-05

**Authors:** Zhouqi Cui, Ching-Hong Yang, Roshni R. Kharadi, Xiaochen Yuan, George W. Sundin, Lindsay R. Triplett, Jie Wang, Quan Zeng

**Affiliations:** 1 Department of Plant Pathology and Ecology, The Connecticut Agricultural Experiment Station, New Haven, Connecticut, United States of America; 2 Department of Biological Sciences, University of Wisconsin-Milwaukee, Milwaukee, Wisconsin, United States of America; 3 Department of Plant, Soil, and Microbial Sciences, Michigan State University, East Lansing, Michigan, United States of America; 4 Department of Energy Joint Genome Institute, Walnut Creek, California, United States of America; University of Florida Institute of Food and Agricultural Sciences, UNITED STATES

## Abstract

Necrotrophic plant pathogens acquire nutrients from dead plant cells, which requires the disintegration of the plant cell wall and tissue structures by the pathogen. Infected plants lose tissue integrity and functional immunity as a result, exposing the nutrient rich, decayed tissues to the environment. One challenge for the necrotrophs to successfully cause secondary infection (infection spread from an initially infected plant to the nearby uninfected plants) is to effectively utilize nutrients released from hosts towards building up a large population before other saprophytes come. In this study, we observed that the necrotrophic pathogen *Dickeya dadantii* exhibited heterogeneity in bacterial cell length in an isogenic population during infection of potato tuber. While some cells were regular rod-shape (<10μm), the rest elongated into filamentous cells (>10μm). Short cells tended to occur at the interface of healthy and diseased tissues, during the early stage of infection when active attacking and killing is occurring, while filamentous cells tended to form at a later stage of infection. Short cells expressed all necessary virulence factors and motility, whereas filamentous cells did not engage in virulence, were non-mobile and more sensitive to environmental stress. However, compared to the short cells, the filamentous cells displayed upregulated metabolic genes and increased growth, which may benefit the pathogens to build up a large population necessary for the secondary infection. The segregation of the two subpopulations was dependent on differential production of the alarmone guanosine tetraphosphate (ppGpp). When exposed to fresh tuber tissues or freestanding water, filamentous cells quickly transformed to short virulent cells. The pathogen adaptation of cell length heterogeneity identified in this study presents a model for how some necrotrophs balance virulence and vegetative growth to maximize fitness during infection.

## Author summary

Virulence and vegetative growth are two distinct lifestyles in pathogenic bacteria. Although virulence factors are critical for pathogens to successfully cause infections, producing these factors is costly and imposes growth penalty to the pathogen. Although each single bacterial cell exists in one lifestyle or the other at any moment, we demonstrated in this study that a bacterial population could accomplish the two functions simultaneously by maintaining subpopulations of cells in each of the two lifestyles. During the invasion of potato tuber, the soft rot pathogen *Dickeya dadantii* formed two distinct subpopulations characterized by their cell morphology. The population consisting of short cells actively produced virulence factors to break down host tissues, whereas the other population, consisting of filamentous cells, was only engaged in vegetative growth and was non-virulent. We hypothesize that this phenotypic heterogeneity allows *D*. *dadantii* to break down plant tissues and release nutrients, while efficiently utilizing nutrients needed to build up a large pathogen population at the same time. Our study provides insights into how phenotypic heterogeneity could grant bacteria abilities to “multi-task” distinct functions as a population.

## Introduction

Based on the modes of nutrition acquisition, plant pathogens can be categorized into biotrophs, necrotrophs, and hemibiotrophs [1, 2]. Obligate biotrophs acquire nutrients from living plant cells, and thus have to maintain host viability. Consequently, biotrophs generally do not produce lytic enzymes and toxins, but rely on sophisticated immune suppression systems to escape host surveillance. Obligate necrotrophs, in contrast, feed on nutrients released from dead or dying cells, and thus have to actively kill host cells and break down host tissues through the production of plant cell wall degrading enzymes (PCWDEs) and toxins. Many pathogens display both biotrophic and necrotrophic phases of nutrient acquisition and are categorized as hemibiotrophs.

*Dickeya dadantii* is the causal agent of bacterial soft rot disease. Although *D*. *dadantii* can survive without producing symptoms during a long time in latent infections, it has a typical necrotrophic phase during the active infection of plants. Infections caused by *D*. *dadanntii* mostly occur on nutrient-rich plant organs, such as tubers, rhizomes, bulbs, and succulent stems and leaves [3]. During infection, *D*. *dadantii* secretes a series of PCWDEs, mainly pectate lyases, to disintegrate the plant cell walls. The type III secretion system (T3SS), a secretion apparatus that injects protein effectors into the host cell, is another necessary virulence factor to elicit cell death in *D*. *dadantii* [4, 5]. Pathogens consume the nutrients released from dead plant cells, and spread to adjoining tubers to incite secondary infections as liquid from the rotting tubers percolates onto others [6].

Under favorable conditions, complete decay of a potato tuber or storage roots can occur within two to three days [3]. One practical challenge for *D*. *dadantii* and many other obligate necrotrophs is how to efficiently consume the large amount of nutrients released from the nutrient-rich organs and use them towards building up the pathogen populations for secondary infection [2, 6]. Efficient nutrient consumption and energy conversion seem to be more important for necrotrophs than for biotrophs and hemibiotrophs, as breaking down of the host epidermis and tissue structures results in the immediate release of nutrients that are available to essentially any microbes in the surrounding environment.

Vegetative growth and virulence expression are two distinct physiological states for bacterial pathogens [7, 8]. Virulence is induced under nutrient limited or stress conditions, whereas vegetative growth occurs under nutrient rich, growth favorable conditions [9, 10]. For example, genes encoding the T3SS are induced when bacteria are cultured under a nutrient limited *hrp*-inducing minimal medium condition but were repressed in nutrient rich media such as Lysogeny Broth [9]. In addition to their different environmental cues, a clear tradeoff has also been observed between virulence and growth in bacteria, as the production of virulence factors compromised the rate of metabolic processes associated with growth [7, 11, 12]. Pathogens have developed complex regulatory systems to sense environmental signals and shift between the two lifestyles upon environmental changes [13]. However, the co-occurrence of both lifestyles in a homogeneous population has not been clearly documented and well understood.

Many bacterial pathogens have been observed to exhibit variable phenotypes in an isogenic population in the host. Heterogeneity is defined as a diversity of phenotypes displayed by isogenic bacteria in similar environments that allow new functionality. Phenotypic heterogeneity increases the flexibility and versatility of bacteria as a population. Phenotypic heterogeneity has been observed in many bacteria in various functions such as antibiotic persistence [14], virulence [10, 15–18], biofilm formation [19], motility [20], and sporulation [21, 22].

In this study, we report that the soft rot pathogen *D*. *dadantii* displays heterogeneity in cell length during infection on potato tubers. Within a clonal population, a subpopulation of *D*. *dadantii* cells formed elongated filamentous cells while the rest of the cells remained short. We show that the proportion of filamentous cells and short cells was affected by infection organs, environmental conditions, stages of infection, and presence of freestanding water. We further demonstrated that filamentous cells and short cells have distinct properties: while short cells were virulent, motile, and relatively tolerant to stress, the filamentous cells were non-virulent, non-motile, and highly sensitive to stress. However, filamentous cells display upregulated metabolic genes and increased growth compared to the short cells. When environmental conditions change, filamentous cells began reverting to short cells. *D*. *dadantii* differentiation into heterogeneous cell types was dependent on the differential expression of the alarmone ppGpp. These findings provide insight into the role of phenotypic heterogeneity in the overall fitness of a bacterial population during host-pathogen interactions.

## Results

### *D*. *dadantii* differentiates into filamentous cells and short cells during the infection of potato tuber.

**Fig 1:**
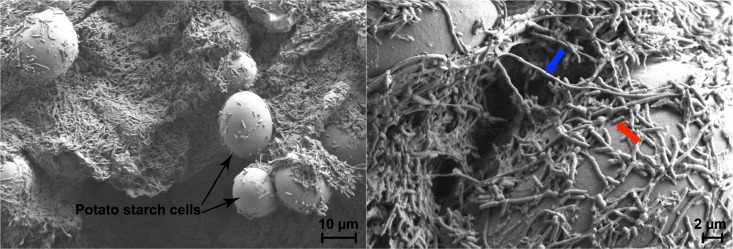
Filamentous cells and short cells of *D*. *dadantii* formed on potato tuber during later stage of infection at two magnifications. Potato tubers were inoculated with a clonal culture of *D*. *dadantii*. The observation was made at 48 hpi at the surface center position of the infected tuber. Black arrow: potato starch cells, blue arrow: a representative filamentous cell (>10 μm), red arrow: a representative short cell (<10 μm).

In our preliminary study of gene expression in single cells of *D*. *dadantii*, we noticed that some *D*. *dadantii* cells presented an elongated cell morphology when inoculated on potato tubers. To determine if *D*. *dadantii* displayed heterogeneity in cell morphology during the infection of potato tuber, tubers inoculated with a clonal culture of wild type *D*. *dadantii* strain 3937 at 48 hrs post inoculation (hpi) were examined under scanning electron microscopy. A heterogeneity in cell length was indeed observed within the isogenic population of *D*. *dadantii*: while some cells were rod-shaped with length between 1 to 10 μm, other cells were highly elongated to lengths greater than 10 μm (Fig 1). The filamentous cells were plated and re-identified to rule out contamination of other non-*Dickeya* species. Hereafter, we define cells shorter than 10 μm as short cells, and cells longer than 10 μm as filamentous cells. Ratio of filamentous cells to short cells range between 1:0.91 to 1:3.83 at 48 hpi.

### *D*. *dadantii* cell filamentation is affected by plant organ, bacterial position in the infection site, time since inoculation, and presence of freestanding water

**Fig 2:**
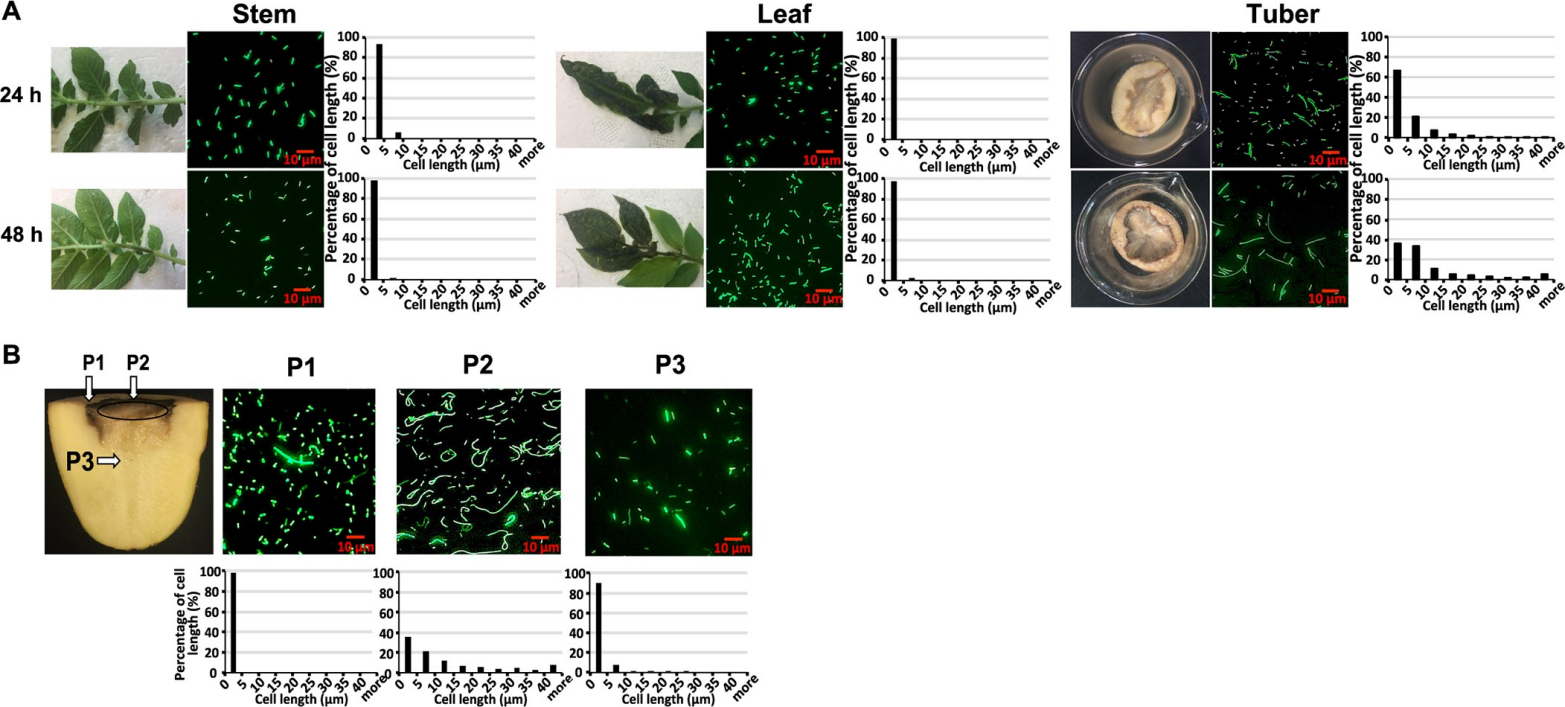
Impact of organ types and infection positions to the distribution of filamentous cells and short cells of *D*. *dadantii* on potato tubers. **(A) Symptoms, cell morphology and length distribution of *D*. *dadantii* at 24 and 48 hpi on potato leaves, stems, and tubers and (B) after 48 hpi on potato tubers sampled at three different positions.** Cells expressing green fluorescence were observed using an epifluorescence microscope. At least three randomly sampled views were analyzed for each sample and one of them was displayed in this figure. The three sampling positions on potato tubers are P1 (surface edge, at the interface of the rotten and healthy tuber tissue), P2 (surface center) and P3 (internal edge, at the interface of the rotten and healthy tuber tissue). Cell length was quantified by ImageJ. The experiment was repeated three times with similar results observed.

Next, we determined whether the formation of filamentous cells observed in potato tubers also occurs in other organ types (leaves and stems). To visualize bacterial cells *in vivo*, we introduced a green fluorescence protein (*gfp*) reporter constitutively expressed under the control of an *nptII* promoter, into the wild type *D*. *dadantii*. We confirmed that expression of *gfp* did not affect cell length (S1 Fig) and used this *gfp-*expressing strain in the characterization hereafter. No obvious cell length differentiation was observed in either nutrient-limited organs such as leaves or stems (filamentous cell percentage <0.3% of total cells) inoculated with *D*. *dadantii* at 24 and 48 hpi, as observed in the nutrient rich organ potato tubers (filamentous cell percentage >17.8% of total cells, Fig 2A).

**Fig 3:**
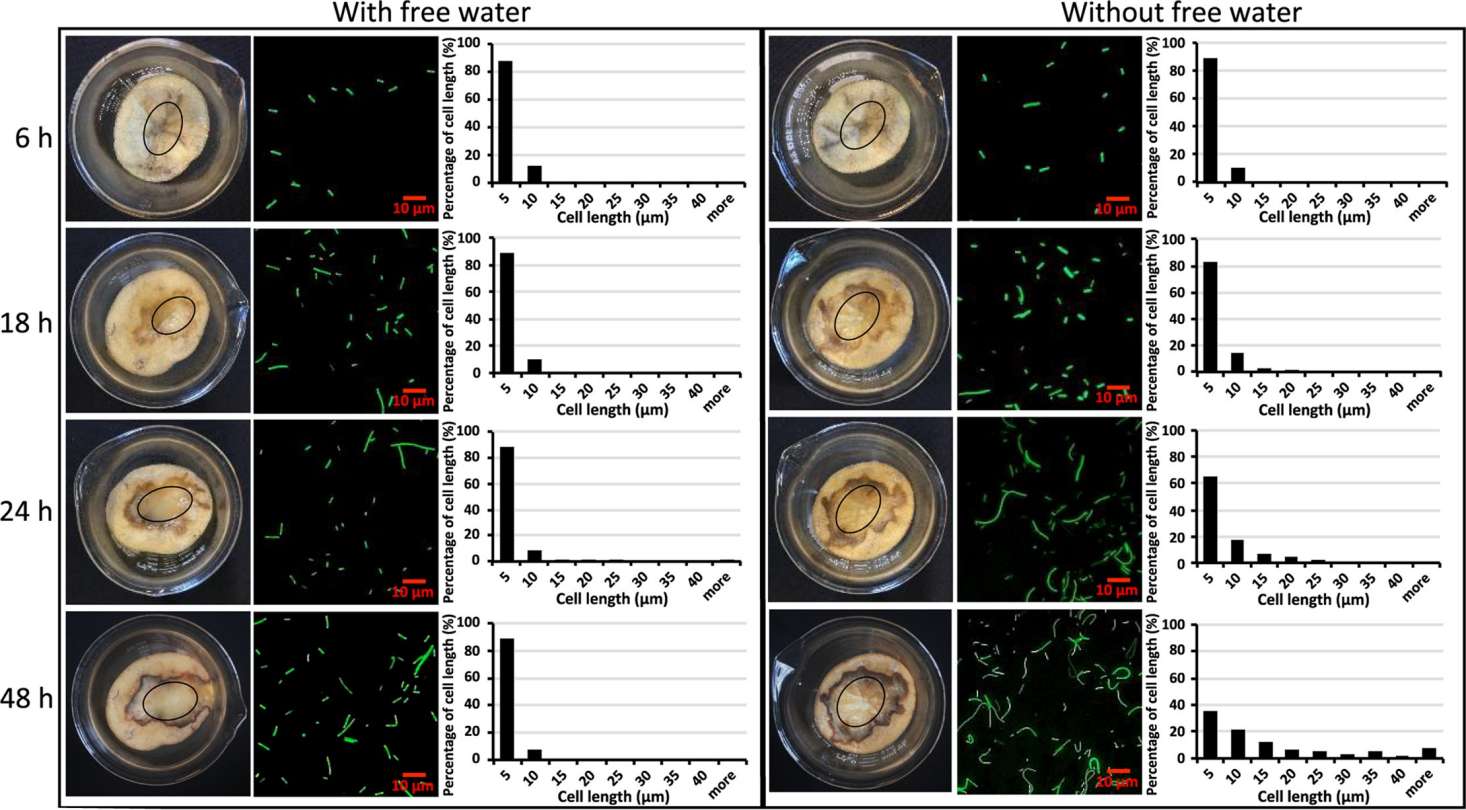
Impact of infection stage and freestanding water on the distribution of filamentous cells and short cells of *D*. *dadantii* on potato tubers. Symptoms, cell morphology and length distribution of *D*. *dadantii* at different stages of infection (6, 18, 24, and 48 hpi) on potato tuber in the presence and absence of freestanding water. Circles on the symptom images represent the sampling area. For potato tubers with freestanding water (left panel), 0.5 ml of sterile distilled water was added to the surface of potato tubers every 4 hr. The experiment was repeated five times with similar results.

We next determined whether the filamentous cell formation could be affected by bacterial position on a potato tuber. Cell lengths of *D*. *dadantii* at the surface edge of infection, at the surface center of infection, and at the internal edge of infection of infected potatoes (P1, P2, and P3 respectively, as indicated in Fig 2B) were quantified at 48 hpi. A significantly higher ratio of filamentous to short cells was observed at the surface center position (P2, 1:1.33), where disease was well established, compared to that observed at the interfaces of healthy and diseased tissues (P1 and P3, 1:399 and 1:37.5) (Fig 2B). Echoing this observation, we also observed a steady increase in the proportion of filamentous cells from an early time point after inoculation (0% of the total population at 6 hpi) to a later time point (42.9% at 48 hpi; Fig 3, right panel).

Water plays an important role in soft rot disease initiation [6]. We observed that the formation of filamentous cells was repressed by the presence of freestanding water on top of the inoculated tubers, with only 3.0% of total cells filamented at 48 hpi, compared with the aforementioned 42.9% of cells filamented without the presence of water (Fig 3). Taken together, these results demonstrate that *D*. *dadantii* forms filamentous cells on potato tubers, at later stages of infection, and at well-established infection positions on the surface (P2). In contrast, the formation of filamentous cells was repressed on leafy tissues, at early infection time points, at disease interfaces (P1 and P3), and on water-covered tissues.

### *D*. *dadantii* cell filamentation is associated with an absence of virulence gene expression

**Fig 4:**
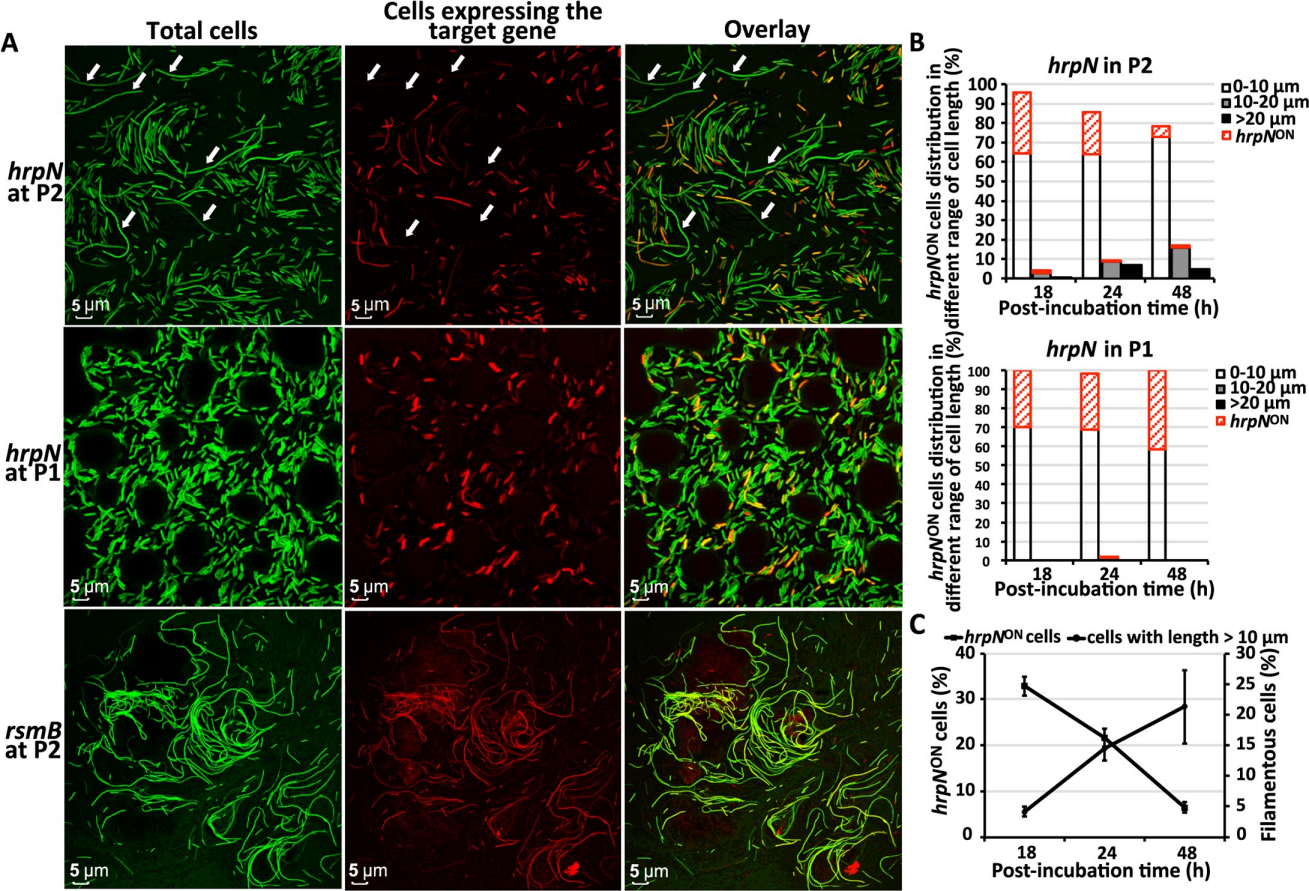
Expression of the T3SS gene *hrpN* in filamentous and short cells of *D*. *dadantii* on potato tuber. **(A) Visualization of *hrpN*-expressing cells (*hrpN*^ON^ cells) in filamentous and short cells of *D*. *dadantii* on potato tuber.** *D*. *dadantii* carrying a *hrpN-mCherry-nptII-gfp* was inoculated onto potato tuber in the absence of freestanding water for 48 h. Potato tissues collected at P1 and P2 positions were observed under confocal microscope. Total bacterial cells were visualized by green fluorescence and *hrpN* ^ON^ cells were visualized by red fluorescence. The expression of a non-T3SS gene *rsmB* (through a *rsmB-mCherry-nptII-gfp* construct) was used as a control. **(B) Percentage of the *hrpN*** ^**ON**^ **cells in filamentous and short cells.** At each time point (18, 24 and 48 hpi), total *D*. *dadantii* cells were categorized into three groups based on their cell length (0–10 μm, 10–20 μm and >20 μm). The proportion of *hrpN* ^ON^ cells within each group is highlighted in red. **(C) Correlation between the percentage of *hrpN*** ^**ON**^ **cells and the percentage of filamentous cells at P2 position at 18, 24, and 48 hpi.** The experiments were repeated three times with similar results.

The observation that most filamentous cells were formed only after infection became well-established i.e., after host cell lysis was underway, suggested that this subpopulation may not need virulence capabilities. We first determined the level of expression of genes encoding the T3SS, a critical virulence factor in *D*. *dadantii*. We introduced a dual fluorescence promoter reporter P*nptII-gfp*-P*hrpN-mCherry* into the wild type *D*. *dadantii* and inoculated it onto potato tuber. The reporter contains two promoter-fluorescence gene fusions, P*nptII*-*gfp* and P*hrpN-mCherry*, which enables visualization of the total population of *D*. *dadantii* (both filamentous cells and short cells) in green fluorescence, while monitoring the expression of *hrpN* in red fluorescence in these two subpopulations. At 48 hpi, a clear differentiation of filamentous cells and short cells was observed at surface center position P2 in the absence of freestanding water (Fig 4A, left panel). Interestingly, a strong negative correlation was observed between cell length and the expression of *hrpN* at the P2 position (Fig 4A, correlation co-efficiency R^2^ = 0.9789). A total of 88.2% of the *hrpN*-expressing cells had a cell length of less than 10 μm and 11.8% of the *hrpN* expressing cells had a cell length between 10 μm and 20 μm. No *hrpN* expression was detected in cells with lengths greater than 20 μm (Fig 4A indicated by arrows, and 4B). At the P1 position, only short cells were observed, and 41.7% of them expressed *hrpN* (Fig 4A and 4B). The expression of a non-T3SS gene, *rsmB*, was evenly expressed in both filamentous and short cells (Fig 4A).

As we determined that the T3SS genes were not expressed in the filamentous cells, and the proportion of filamentous cells increased as the infection progressed from early to later stage at P2 position (Fig 3 without freestanding water), next, we determined whether the increase of the filamentous cell subpopulation would result in an overall reduction of the T3SS expression at P2 position. As shown in Fig 4C, filamentous cell subpopulation increased from 4.0% at 18 hpi to 18.6% at 48 hpi at P2 position. At the same time, a decrease in T3SS expression was also indeed observed (from 33.0% of total cells at 18 hpi to 6.8% of the total cells at 48 hpi, Fig 4C). No increase in filamentous cell proportion nor decrease of the T3SS expressing cells in the total population was observed at the disease interface P1 (Fig 4B).

**Fig 5:**
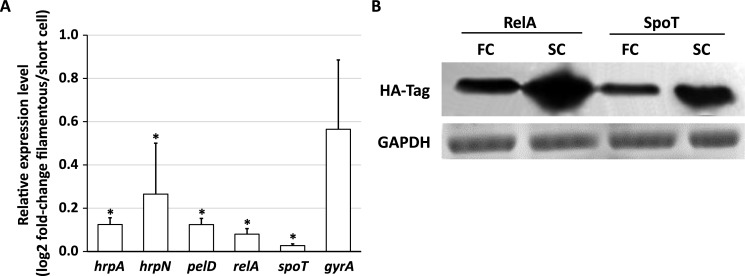
Expression of virulence and ppGpp biosynthesis genes in the filamentous and short cells of *D*. *dadantii*. **(A) Relative mRNA abundance of *hrpA*, *hrpN*, *pelD*, *relA*, *spoT* and *gyrA* (filamentous cells/short cells) quantified by qRT-PCR. (B) Western blot analysis of RelA and SpoT protein levels in filamentous and short cells** *D*. *dadantii* filamentous and short cells were obtained from the P2 position of infected potato tubers after 48 h of incubation. The short cells and the filamentous cells were separated by sucrose gradient centrifugation as illustrated in S2 Fig. *rplU* was used as an endogenous control for qRT-PCR. Error bars indicate standard errors of the means. Asterisks indicate statistically significant differences of the means (*P* < 0.05 by Student’s *t* test). HA tag was inserted at the 3’ end of the ORFs of *relA* and *spoT*. RelA and SpoT proteins were detected by α-HA antibody. GAPDH stained with Coomassie blue was used as the loading control. The experiments were repeated three times with similar results.

To further confirm the differential expression of T3SS genes in filamentous cells and short cells, we developed a sucrose-gradient centrifugation method to separate enriched pools of filamentous cells and short cells from position P2 of the infected potato tubers (S2 Fig). Expression of two T3SS marker genes, *hrpN* and *hrpA*, was analyzed in the two subpopulations by qRT-PCR. As shown in Fig 5A, greater than 75% reduction of the expression of both genes was observed in filamentous cells compared to short cells, further supporting the hypothesis that filamentous cells express T3SS genes at a reduced level. We next compared the mRNA levels of another virulence factor, the pectate lyase gene *pelD*, in the two subpopulations. Compared to the short cells, the mRNA of *pelD* is more than fivefold reduced in the filamentous cells. No significant difference in the expression of *gyrA*, a gene encoding DNA gyrase, was detected between the two subpopulations (Fig 5A). Together, our results suggest that filamentous cells, mostly formed when disease is well-established, may not participate in virulence and host invasion.

### Alarmone ppGpp biosynthesis genes *relA* and *spoT* are differentially expressed in filamentous cells and short cells of *D*. *dadantii*

Previous studies have suggested a role of alarmone ppGpp in determining bacterial cell length; mutation of ppGpp biosynthesis genes resulted in elongated cell morphology in *Erwinia* and *Pseudomonas* plant pathogens [23, 24]. However, it is unclear whether the formation of naturally occurring filamentous cells could be caused by the differential expression of the ppGpp biosynthesis genes. To determine the role of ppGpp in filamentous cell formation in *D*. *dadantii*, we measured the mRNA levels of two putative ppGpp biosynthesis genes, *relA* and *spoT*, in the separated short cells and enriched filamentous cells collected from infected potato tubers. Expression of both genes was significantly reduced in the filamentous cells compared to the short cells (Fig 5A). To validate this differential expression, a Western blot was performed to quantify the RelA and SpoT proteins in the two subpopulations. Similar to the qRT-PCR results, higher levels of RelA and SpoT proteins were detected in the short cells compared to that in the filamentous cells (Fig 5B). The above results conclude that the two putative ppGpp biosynthesis genes are differentially expressed in filamentous cells and short cells of *D*. *dadantii*.

### Double deletion of *relA* and *spoT* abolishes intracellular ppGpp levels and induces cell elongation in *D*. *dadantii*

The reduction in *relA* and *spoT* expression in filamentous cells led us to hypothesize that *relA* and *spoT*-mediated ppGpp production is essential for *D*. *dadantii* filamentation on potato tubers. First, we confirmed whether *relA* and *spoT* are required for ppGpp biosynthesis and degradation in *D*. *dadantii* as in other bacteria. Deletion mutants of *relA* and *spoT* were generated and intracellular ppGpp levels were measured. Compared to the wild type, decreased and increased ppGpp levels were observed in Δ*relA* and Δ*spoT* respectively, and could be restored through complementation (Table 1). In the Δ*relA*Δ*spoT* double mutant, drastic reduction of ppGpp levels were observed (Table 1). These observations suggest that as in model species of bacteria, *D*. *dadantii* RelA is responsible for ppGpp synthesis, while SpoT has a major function of ppGpp hydrolysis as well as a minor function of ppGpp synthesis.

**Table 1:** Relative intracellular ppGpp level of ppGpp biosynthesis gene mutants andcomplementation strains.

	WT	Δ*relA*	Δ*relA* pCL*relA*	Δ*spoT*	Δ*spoT* pCL*spoT*	Δ*relA*Δ*spoT*
Intracellular ppGpp level relative to WT	1±0.04	0.89±0.02	1.78±0.06*	3.83±0.21*	0.70±0.10	0.49±0.02*

* denotes significant difference (*p <* 0.05).

pCL*relA* and pCL*spoT* are pCL1920 plasmids carrying *relA* and *spoT* respectively, used for complementation purposes.

**Fig 6:**
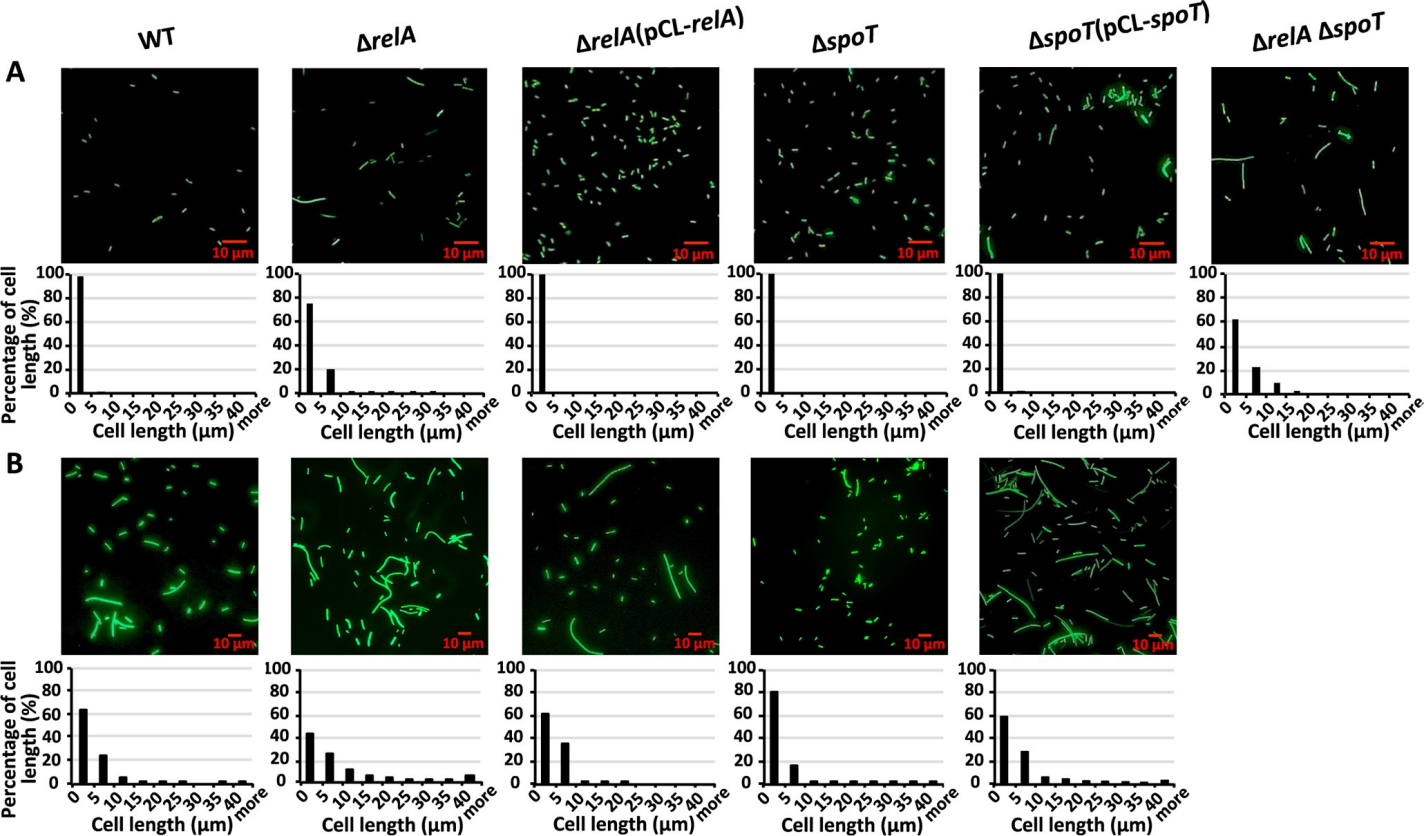
Impact of ppGpp on cell length of *D*. *dadantii*. **Morphology and quantification of cell length of wild type, ppGpp biosynthesis gene mutants and the complementation strains at 18 h in LB broth (A) and at the surface center (P2) position of potato tuber in the absence of freestanding water 48 hpi (B).** Δ*relA* Δ*spoT* mutant strain is non-virulent on potato tuber thus the cell length data of Δ*relA* Δ*spoT* on potato tuber is unavailable. The experiments were repeated three times with similar results.

We observed that filamentous cells formed under natural infection conditions exhibited reduced expression of both *relA* and *spoT*. To determine whether these genes are necessary for the filamentous cell formation in *D*. *dadantii*, we compared the cell length of wild type, Δ*relA*Δ*spoT*, single deletion mutants and complementation strains in culture (LB broth) and on potato tuber. Cell length was significantly increased when both ppGpp biosynthesis genes were mutated (Δ*relA*Δ*spoT* in Fig 6A). We also observed a strong negative correlation between a strain’s intracellular ppGpp levels (Table 1) and cell length; cell length was increased in deletion mutants with reduced intracellular ppGpp levels (Δ*relA* and Δ*relA*Δ*spoT*, Fig 6A) and was decreased in a deletion mutant with increased ppGpp level (Δ*spoT*, Fig 6A). On potato tuber, a similar correlation was also observed (Fig 6B), although the cell length of Δ*relA*Δ*spoT* could not be determined, as the double mutant was non-virulent. These observations further confirmed that intracellular ppGpp strongly affects bacterial cell length, and abolishing ppGpp synthesis through double mutation of *relA* and *spoT* results in filamentous cell formation similar to that observed in potato tuber.

To confirm that ppGpp itself and not another function of RelA or SpoT is a signal for cell elongation, we next tested the effect of adding chemically synthesized ppGpp on filamentous cell formation. We hypothesized that if ppGpp causes elongation and has limited permeability [25], adding chemically synthesized exogenous ppGpp at a concentration much higher than the intracellular ppGpp concentration to *D*. *dadantii* would repress filamentous cell formation. To test this hypothesis, ppGpp at two different concentrations was added on top of the potato tubers inoculated with wild-type *D*. *dadantii*. A significant reduction in the proportion of filamentous cells was observed upon addition of ppGpp (S3 Fig), which further supported the hypothesis that low ppGpp triggers filamentous cell formation during *D*. *dadantii* infection.

### Filamentous cells induced by ppGpp deficiency do not express virulence factors and do not participate in pathogenicity during host-microbe interactions

**Fig 7:**
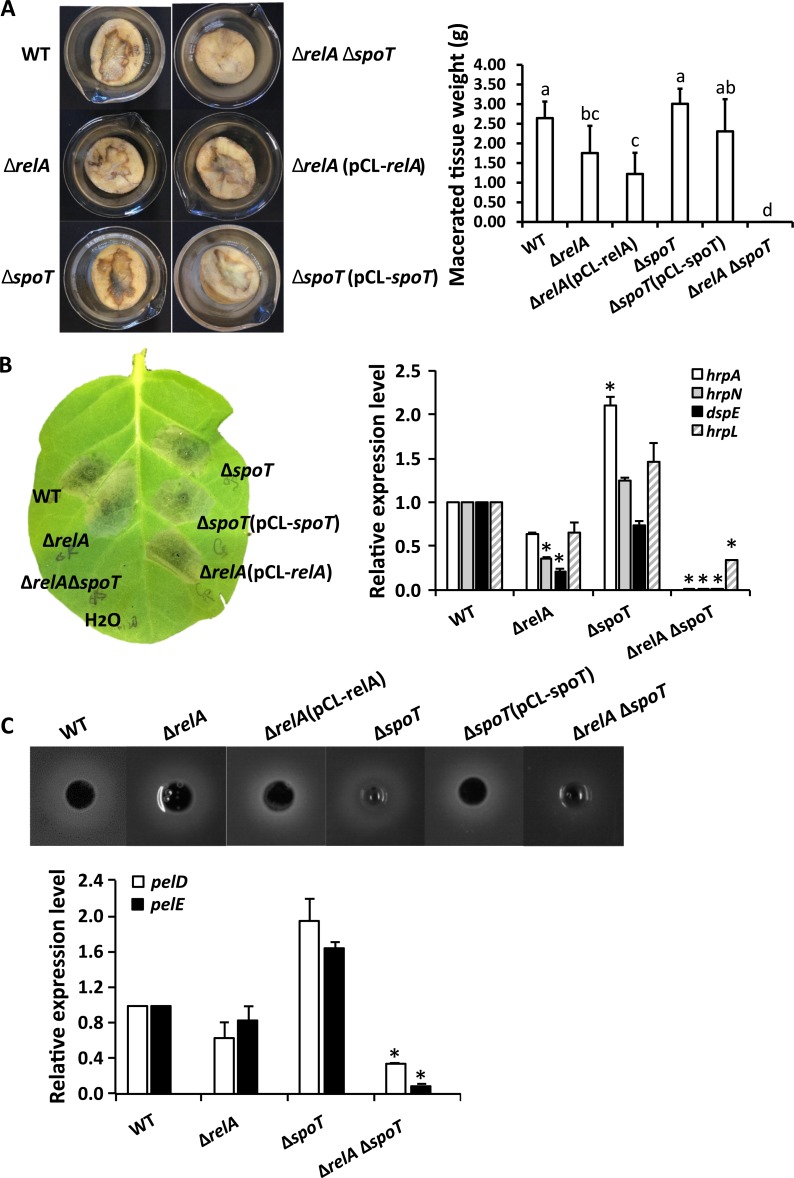
**Impact of ppGpp on virulence in *D*. *dadantii*. Disease symptoms and quantification on potato tuber (A), hypersensitive response (or induced cell death) on tobacco leaf and expression of T3SS genes in *hrp-*inducing minimal medium (B), and Pel production and expression of *pel* genes (C) in wild type, ppGpp biosynthesis mutants, and the complementation strains.** The pathogenicity assay was performed on potato tubers in the absence of free-standing water, with symptoms documented at 18 hpi; the hypersensitive response was performed by infiltrating the bacterial suspension (10^8^ CFU/ml) into tobacco leaves, with symptoms documented at 20 hpi. The Pel production was measured in cells cultured in Pel-MM plates for 20 hrs. The expression of T3SS genes and *pel* genes was measured by qRT-PCR in cells cultured in *hrp*-inducing MM and *pel*-MM for 12 hrs, respectively. Presence of different letters indicates significant difference (*P* < 0.05). The experiments were repeated three times with similar results.

Having confirmed that cell length heterogeneity is dependent on the differential expression of ppGpp biosynthesis genes, and that filamentous cells can be artificially induced through deletion of both *relA* and *spoT*, we used the double deletion Δ*relA*Δ*spoT* mutant to uncover putative biological functions of the naturally occurring filamentous cells. Naturally occurring filamentous cells exhibited significantly reduced expression of the T3SS and pectate lyase genes, which indicates that these cells may not participate in pathogenicity during the host-microbe interaction. To test this hypothesis, we compared the bacterial virulence of wild type and Δ*relA*Δ*spoT*, along with single mutants and complementation strains, on potato tubers. Compared to the wild type which caused necrotic lesions on potato tuber at 18 hpi, Δ*relA*Δ*spoT* lost its virulence capability (Fig 7A).

To determine whether the reduction in T3SS and pectate lyase gene expression in the naturally occurring filamentous cells would also result in reduced activities of T3SS and of pectate lyases, we compared the hypersensitive response (HR) and pectate lyase production in wild type *D*. *dadantii* and the Δ*relA*Δ*spoT* double mutant, as well as single mutants and complementation strains. Compared to the wild type, Δ*relA*Δ*spoT* lost its ability to elicit the HR on tobacco leaves (Fig 7B). Consistent with phenotypic observations, mRNA levels of the T3SS genes *hrpA*, *hrpN*, *dspE*, and *hrpL* were also significantly reduced in Δ*relA*Δ*spoT* as observed in naturally occurring filamentous cells under the *in vitro hrp-*inducing condition (Fig 7B). A significant reduction in pectate lyase production and in expression of pectate lyase-encoding genes *pelD* and *pelE* was also observed in Δ*relA*Δ*spoT* compared with the wild type under *in vitro* Pel producing conditions (Fig 7C). Taken together with the results from Fig 4, these results suggest that whether environmentally or mutationally induced, filamentous cells of *D*. *dadantii* do not produce two essential virulence factors and do not contribute to pathogenicity.

### Filamentous cells are non-motile

**Fig 8:**
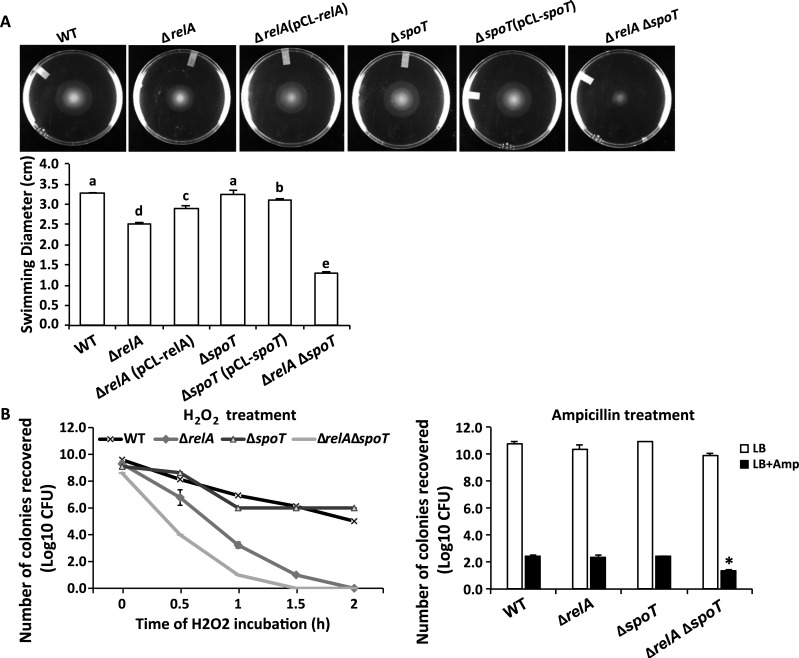
Impact of ppGpp on motility and stress tolerance of *D*. *dadantii*. **(A)** Swimming motility of the wild-type, ppGpp biosynthesis mutants and the complementation strains after 19 hpi. Presence of different letters indicates significant difference (*P* < 0.05). **(B)** Survival of wild type and ppGpp biosynthesis mutants after exposed to 10mM hydrogen peroxide for 0–2 h (left panel) and after plating on LB agar or LB agar supplemented with 1 μg ml^-1^ ampicillin for 48 h (right panel). Presence of asterisk indicates significant difference (*P* < 0.05). The experiments were repeated three times with similar results.

Motility is essential for *D*. *dadantii* to spread and cause secondary infections, especially under wet conditions [26]. Our observation that *D*. *dadantii* filamentous cell formation only occurred in the absence of freestanding water suggests that filamentous cells may be non-motile. To test this hypothesis, a mixture of filamentous and short cells collected at the P2 position of potato tubers at 48 hpi were resuspended in sterile distilled water and observed under the microscope for their motility (S1 Video). As expected, filamentous cells showed significantly reduced motility (0.723 ± 0.6 microns per second) compared to short cells (5.508 ± 2.621 microns per second). We also compared the swimming and swarming motility of wild type *D*. *dadantii* with deletion mutants of the ppGpp biosynthesis genes. A complete abolishment of both swimming and swarming motility was observed in Δ*relA*Δ*spoT* (Fig 8A and S4 Fig). These results demonstrate that filamentous cells are non-motile. It also indicates that ppGpp biosynthesis is required for bacterial motility in *D*. *dadantii*.

### Filamentous cells induced by ppGpp deficiency are more sensitive to hydrogen peroxide and ampicillin stress

Previous studies documented that some bacteria form filamentous cells as a response to stress e. i. antibiotic persistence or host immune response [27]. To determine whether filamentous cells of *D*. *dadantii* serve the same purposes, we compared the survival of double and single deletion mutants of ppGpp biosynthesis genes with the wild type upon two stress conditions hydrogen peroxide (H_2_O_2_) and antibiotic ampicillin treatment. Compared to the wild type, Δ*relA*Δ*spoT* displayed increased susceptibility to sub-lethal dosage of both hydrogen peroxide and ampicillin (Fig 8B). These results suggest that, in contrast to previous examples, mutationally induced filamentous cells of *D*. *dadantii* not only do not grant the bacteria any advantage, but rather compromised their ability, in tolerating H_2_O_2_ and antibiotic ampicillin. The observation that filamentous cells are more sensitive to oxidative stress is also in agreement with our observation that filamentous cells are more abundantly present at later stage of infection, when the host cells are well decayed and host defense is significantly compromised.

### Filamentous cells are associated with increased expression of growth and metabolism-associated genes and faster growth compared to short cells on decayed potato tubers

**Fig 9:**
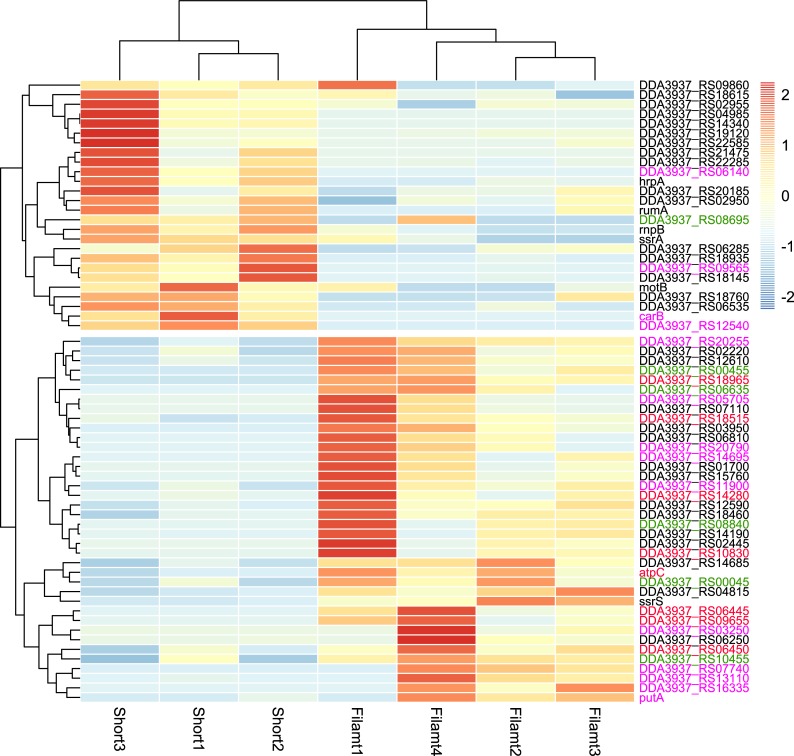
Genes differentially expressed in filamentous cells and short cells identified by RNA-seq. Relative mRNA abundance in each sample was indicated by color. Gene IDs were color-coded into the following categories: transportation (green); respiration (red); metabolism (magenta).

Despite finding that filamentous cells do not participate in virulence, are non-motile, and more sensitive to stress, we sought clues as to the fitness advantages of these filamentous cells during the host-microbe interactions. To further elucidate the biological functions, a transcriptomic analysis was performed in the separated short cells and enriched filamentous cells collected from potato tubers at 48 hpi (S2 Fig). Compared to the short cells, 27 and 38 genes were found to be down-regulated and up-regulated in the filamentous cells, respectively (Fig 9 and Table 2). Among the down-regulated genes (*p <* 0.05), genes encoding the T3SS (*hrpA*), motility (*motB*) and stress tolerance (RS18760 and *dprA*) were identified. Down regulation of ppGpp biosynthesis genes *relA* (relative mRNA expression filamentous cells/short cells = 0.43) and *spoT* (0.85), as well as a few pectate lyase and T3SS genes (*pelE*, *hrpY* and *hrcV*; relative mRNA expression filamentous cells/short cells = 0.23, 0.46, and 0.09 respectively) were also identified, although the difference was not statistically significant (*p* >0.05), possibly due to the fact that the enriched filamentous cell samples still contained mixed-in short cells (S2 Fig). Among the up-regulated genes, surprisingly, over 60% (23/38) of them are related to bacterial growth. Specific function categories included substrate transport (5 genes), respiration (8 genes), and metabolism (10 genes) (Fig 9 and Table 2).

**Table 2:** Genes with altered expression in filamentous cells compared to short cells identified in RNA-seq.

Gene ID	log2 Fold Change	p-value	Category	Function
**Genes that are down-regulated in filamentous cells**				
DDA3937_RS18145	-5.72	3E-07		hypothetical protein
DDA3937_RS18935	-4.70	4E-04		N/A
DDA3937_RS09565	-4.27	3E-03	Metabolism	translation initiation factor IF-1
*carB*	-4.25	0.02	Metabolism	carbamoyl-phosphate synthase large subunit
DDA3937_RS12540	-4.24	0.02	Metabolism	phosphatidylglycerophosphatase B
DDA3937_RS04985	-4.03	0.04	Protease	pitrilysin
DDA3937_RS14340	-4.03	0.04	Regulation	transcriptional regulator PecT
DDA3937_RS06140	-3.99	0.05	Metabolism	lipoate synthase
DDA3937_RS21475	-3.97	0.05		hypothetical protein
DDA3937_RS22585	-3.48	0.05		hypothetical protein, second of an operon
DDA3937_RS06535	-3.31	0.02	tRNA	tRNA-Lys
*hrpA*	-3.29	3E-04	Virulence	Type III secretion system pilus
*motB*	-2.72	0.05	Motility	motility protein MotB
*rumA*	-2.73	0.03	rRNA	23S rRNA (uracil(1939)-C(5))-methyltransferase RlmD
DDA3937_RS12785	-2.62	0.03		hypothetical protein
DDA3937_RS08695	-2.59	0.05	Transportation	EamA/RhaT family transporter
DDA3937_RS19120	-2.36	0.03	Stress response	DNA-protecting protein DprA
DDA3937_RS18760	-2.32	0.05	Stress response	Organic hydroperoxide resistance transcriptional regulator
DDA3937_RS22285	-2.26	1E-04		hypothetical protein
DDA3937_RS09860	-2.09	0.03		Right-handed parallel beta-helix repeat-containing protein
DDA3937_RS18615	-1.86	0.02	tRNA	tRNA (adenosine(37)-N6)-dimethylallyltransferase MiaA
DDA3937_RS06285	-1.80	0.05	tRNA	tRNA-Leu
DDA3937_RS02950	-1.69	0.02	DNA primase	DNA primase
DDA3937_RS20185	-1.67	0.04		DUF413 domain-containing protein
DDA3937_RS02955	-1.66	0.01	Translation	30S ribosomal protein S21
*rnpB*	-1.61	5E-08	RNA degradation	RNase P RNA component class A
*ssrA*	-1.11	3E-07	tRNA	Transfer-messenger RNA
**Genes that are up-regulated in filamentous cells**				
DDA3937_RS10455	1.08	0.04	Transportation	Channel-type Transporters;Beta barrel porins (The Outer Membrane Porin (OMP)
DDA3937_RS02220	1.10	0.02	Microbe-insect interaction	Delta-endotoxin CytB
DDA3937_RS06450	1.36	0.02	Respiration	Succinate dehydrogenase flavoprotein subunit
DDA3937_RS11900	1.41	5E-03	Metabolism	L-threonine dehydrogenase
DDA3937_RS18460	1.44	0.04	Regulation	Energy-dependent translational throttle protein EttA
DDA3937_RS14685	1.80	0.04	Secondary metabolite	Non-ribosomal peptide synthetase
DDA3937_RS12590	1.84	0.02		Glycine zipper 2TM domain-containing protein
DDA3937_RS12610	2.10	0.03		Monothiol glutaredoxin, Grx4 family
*atpC*	2.12	0.01	Respiration/Energy metabolism	F0F1 ATP synthase subunit epsilon
DDA3937_RS18515	2.12	0.02	Glycolysis/respiration	Phosphoglycerate kinase
DDA3937_RS00045	2.26	0.04	Transportation of ribose	Ribose ABC transporter substrate-binding protein RbsB
DDA3937_RS20255	2.37	1E-03	Metabolism	Acetyl-CoA C-acyltransferase FadA
DDA3937_RS04815	2.49	8E-03		Alpha/beta hydrolase
*putA*	2.59	0.04	Metabolism	Trifunctional transcriptional regulator/proline dehydrogenase/L-glutamate gamma-semialdehyde dehydrogenase
DDA3937_RS14280	2.95	0.03	Respiration/Energy metabolism	NADH:ubiquinone oxidoreductase membrane subunit M
DDA3937_RS06250	3.56	7E-03	tRNA	tRNA (N6-isopentenyl adenosine(37)-C2)-methylthiotransferase MiaB
*ssrS*	3.60	2.15E-10		Unknown
DDA3937_RS13110	3.77	0.02	Metabolism	Beta-ketoacyl-[acyl-carrier-protein] synthase II
DDA3937_RS06445	3.95	7E-03	Respiration/citric acid cycle	Succinate dehydrogenase, membrane subunit, binds cytochrome b556
DDA3937_RS08840	4.01	0.05	Transportation	MFS transporter
DDA3937_RS05705	4.01	0.05	Metabolism	Acyl-CoA thioesterase II
DDA3937_RS07110	4.01	0.05		DUF1611 domain-containing protein
DDA3937_RS02445	4.07	0.04		Peptide chain release factor 3
DDA3937_RS16335	4.08	0.04	Metabolism	Aldehyde dehydrogenase EutE
DDA3937_RS20790	4.10	0.04	Metabolism	Acyl-CoA dehydrogenase
DDA3937_RS07740	4.15	0.03	Metabolism	Siderophore biosynthesis protein SbnG
DDA3937_RS10830	4.16	0.03	Respiration	Serine 3-dehydrogenase
DDA3937_RS01700	4.19	0.03	DNA replication	DnaA initiator-associating protein DiaA
DDA3937_RS14190	4.22	3E-03		Membrane protein
DDA3937_RS03250	4.27	0.03	Metabolism	Glycine cleavage system protein H
DDA3937_RS06810	4.32	0.02		Hypothetical protein
DDA3937_RS03950	4.34	0.01		DNA sulfur modification protein DndD
DDA3937_RS14695	4.34	0.02	Metabolism	Chrysobactin oligopeptidase cbsH, hydrolizes chrysobactin,
DDA3937_RS06635	4.71	5E-03	Transportation	Molybdate ABC transporter substrate-binding protein
DDA3937_RS09655	4.80	4E-03	Respiration	Formate C-acetyltransferase
DDA3937_RS15760	4.93	1E-03	Regulation	rseA (anti-sigma factor)
DDA3937_RS18965	5.06	5E-04	Respiration	Fumarate reductase (quinol) flavoprotein subunit
DDA3937_RS00455	5.07	5E-04	Transportation	Malate permease

**Fig 10:**
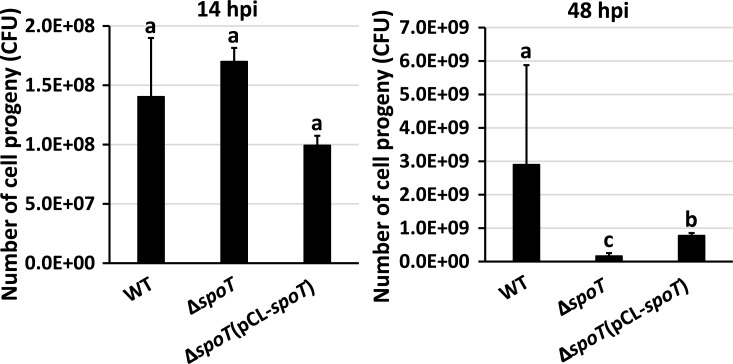
The number of the progenies produced by *D*. *dadantii* wide type, Δ*spoT*, and Δ*spoT* (pCL*-spoT*). Bacteria were inoculated to potato tubers in the absence of freestanding water. After 14 and 48 hpi, 0.03 gram of potato tissue was collected from the P2 position. The tissues were resuspended in 10ml of water and incubated for one hour at 28°C to induce short cell formation. Presence of different letters (a, b, and c) indicates significant difference (*P* < 0.05). The experiments were repeated four times by two independent persons with similar results.

To determine if the higher rate of metabolism in filamentous cells would result in faster growth on potato tuber, we compared the number of progenies produced by the wild type, which formed both filamentous cells and short cells, and by Δ*spoT*, which only formed short cells (Fig 6A). Forty-eight hours after inoculation, 2.9×10^9^ CFU of wild type were recovered per 0.03 gram of potato tissue after incubation in water to induce the short cells (Fig 10, 48 hpi). Although Δ*spoT* showed no attenuation in virulence compared to the wild type, it only produced 1.7×10^8^ CFU of cells from the same amount of tissue within the same period of time, or approximately 6% of the wild type (Fig 10, 48 hpi). This reduction in growth in Δ*spoT* could be partially restored to the wild-type level through complementation (7.8×10^8^ CFU). Interestingly, such growth difference between wild type and Δ*spoT* was not observed at earlier stage of infection when filamentous cells were not formed (Fig 10, 14 hpi). These data suggest that filamentous cells formed at later stage of infection granted *D*. *dadantii* faster growth on potato tuber.

### Filamentous cells can transform to short cells upon exposure to fresh potato tissues and freestanding water

**Fig 11:**
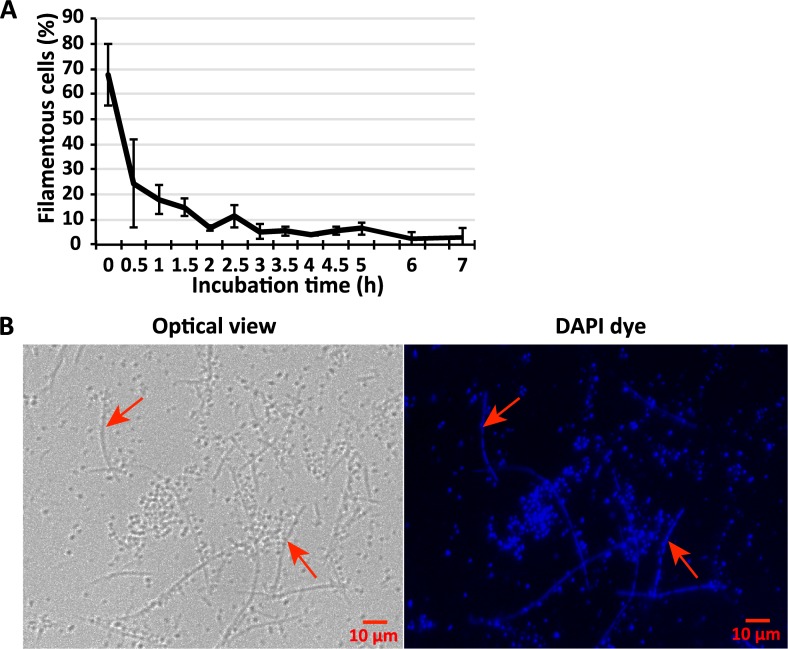
**(A) Percentage of filamentous cells (>10** μ**m) of *D*. *dadantii* upon exposure to fresh potato tuber tissues for different period of time.** The filamentous cells were collected from the surface center (P2) of an infected potato tuber slide after 48 hpi, and transferred onto a freshly dissected potato tuber. **(B) DAPI staining of the nucleoids of *D*. *dadantii* collected from the surface center (P2) of the infected potato tuber in the absence of freestanding water, at 48 hpi.** The representative filamentous cells, which contained multiple, segmented nucleoids, were indicated by arrows. The experiments were repeated three times with similar results.

Filamentous cells contain large amount of biomass and may have the potential to split into short cells. To test this hypothesis, a mixture of filamentous cells and short cells collected from the P2 position of a decayed potato after 48 hpi were transferred to a fresh potato tuber. The percentage of filamentous cells and short cells were monitored after the transfer. As expected, exposure to fresh potato tissue caused a drastic reduction in filamentous cell percentage in the total population, from 67.9% at 0 hpi, to 18.0% at 1 hpi, and only 3.9% at 4 hpi (Fig 11A). To visualize the transformation process, a time-lapse movie was captured documenting long, filamentous cells dividing to form multiple shorter cells upon exposure to freestanding water (S2 Video). As additional evidence, we used DAPI to stain the nucleoids of the filamentous cells. Multiple segmented nucleoids were observed in some of the filamentous cells, suggesting that filamentous cells have the potential to divide into short cells (Fig 11B). All together, these data support that the non-virulent filamentous cells may be transformed to virulent short cells when environmental conditions are suitable for infection and/or spread.

## Discussion

In this study, we report heterogeneity in the morphology and physiology of a genetically homogeneous population of *D*. *dadantii* during host infection. We determined that *D*. *dadantii* adopts a bistable state in the host in which subpopulations adopt a suite of multiple coordinated phenotypes: short cells were associated with virulence gene expression, slower growth, and active motility, while filamentous cells were associated with lack of virulence gene expression, faster growth, reduced stress tolerance, and lack of motility. Furthermore, we found that the alarmone signal molecule ppGpp is critical for the differentiation of cells into the two subpopulations.

The phenotypic states we observed were most obviously characterized by differentiation in cell morphology and in virulence. Bacterial elongation and filamentation under natural conditions have been previously reported in a few bacteria [27–30], with proposed biological roles mainly related to stress tolerance [27]. For example, upon detection of host immune activation, subpopulations of uropathogenic *Escherichia coli* (UPEC) become filamentous during acute infection in the oligotrophic bladder environment [29]. Filamentous cells are more resistant to neutrophil phagocytosis, and are thought to play a role in the survival and growth of UPEC during maturation of the intracellular bacterial communities (IBC) [30]. In other organisms, bacterial filamentation was shown to confer tolerance to protist gazing [31] and antibiotic exposure [32, 33]. Different from the previous studies, here we showed that filamentous cells of *D*. *dadantii* are not involved in tolerance of the two stress conditions tested. Instead, they were induced in a nutrient-rich host tissue when infection was well established, possibly as a mechanism to maximize pathogen growth and nutrient utilization. While we observed that filamentation may be associated with increased growth capacity and expression of metabolic genes, the adaptive benefit to cell elongation itself remains to be seen.

**Fig 12:**
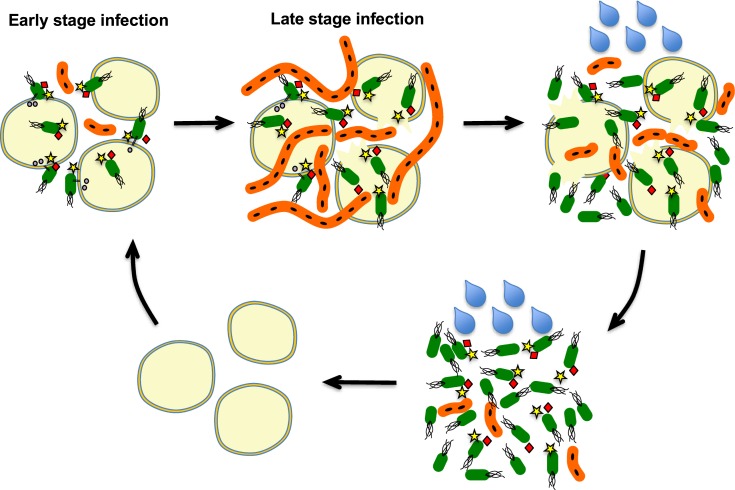
Illustration of the model for the *D*. *dadantii* short cell / filamentous cell differentiation and their respective functions during the infection of potato tuber. At the early stage of infection, the majority of *D*. *dadantii* cells are in the short cell morphology (green colored). These short cells produce pectate lyases (diamonds and stars) to disintegrate plant cell wall and secretes type III effector proteins (circles) to induce host cell death. As infection progresses, many plant cells are killed and nutrients are released, a subpopulation of filamentous cells starts to emerge (orange-colored). These filamentous cells do not produce any virulence factors, do not have motility, and are relatively sensitive to environmental stress. However, these cells have much faster metabolism than the short cells, which enable them to more efficiently consume the nutrients released and build up a large population. When environmental conditions favor infection or spread (e.g. when freestanding water is present), the filamentous cells which contain multiple nucleoids, convert back to short cells and cause secondary infection.

Heterogeneity may provide fitness advantages for a bacterial population as a whole [34, 35]. Heterogeneity in virulence expression has been observed in other bacterial pathogens [10, 11, 17], where it was observed that expression of virulence genes imposes a penalty on bacterial growth rate [11]. However, the best-studied example of physiological heterogeneity is the phenomenon of antibiotic persistence. Persistence is a strategy in which a subpopulation of cells (persisters) exists in a dormant state that confers increased tolerance to environmental stress [36]. Here heterogeneity is thought to work as a “bet-hedging” strategy, benefiting the population as a whole by increasing its adaptability to changing environments. It was also observed that persister cell formation is enhanced by nutrient limitation and other sources of cellular stress [37, 38]. In contrast to persistence, we found evidence that a subpopulation of *D*. *dadantii* forms filaments and increases its growth capacity in high-nutrient tissues, but this comes at a cost of virulence gene expression and tolerance to certain stresses. We hypothesize that in *D*. *dadantii*, phenotypic heterogeneity may allow the pathogen to simultaneously accomplish the two conflicting goals of virulence and vegetative growth. This is has been called a “division of labor” strategy of phenotypic heterogeneity, in which a population benefits from two physiological states with different tradeoffs. A proposed model for the occurrence and function of phenotypic heterogeneity during the *D*. *dadantii* infection cycle is described in Fig 12.

Interestingly, the alarmone signal ppGpp was implicated in a positive role in the induction of the persister state [39]. Evidence points to stochastic noise in ppGpp fluctuation as the most likely driver in the transition between persister and nonpersister states in individuals of a bistable bacterial population [40]. Here we observed a strong negative relationship between ppGpp and the filamentation of *D*. *dadantii* in tubers, finding that ppGpp biosynthesis genes are differentially expressed in filamentous and short cells, that exogenous application of ppGpp shifts the proportion of filamentous cells, and that filamentation phenotypes can be recapitulated through the deletion of ppGpp biosynthesis genes. In light of these findings, we propose that ppGpp variation also mediates the multi-phenotype bistable state observed in *D*. *dadantii* during host infection. ppGpp regulates diverse phenotypes including virulence, growth and motility [39, 41, 42], thus cell-to-cell variation in ppGpp concentration could drive the tandem expression of multiple phenotypes in single cells of *D*. *dadantii*. In linking ppGpp to a second form of bistable state marked by coordinated cell length, virulence, and metabolic traits, this study suggests that ppGpp-mediated pathogen population heterogeneity may be more diverse and significant under native infection conditions than previously recognized. This bistable state we observed was specific to certain conditions of host tissues, infection sites, and water availability levels; the environmental factors and additional mechanisms that trigger conditional bistability is unknown. However, these findings raise the possibility that similar phenomena are occurring in other pathogens that do not undergo such easily observed morphological changes during infection.

## Materials and methods

### Bacterial strains, plasmids, primers and media

Bacterial strains, plasmids, and oligonucleotide primers used in this study are listed in S1 Table. Strains were stored at -80°C in 20% glycerol. *Dickeya dadantii* strains were cultured in Lysogeny Broth (LB) medium, mannitol-glutamic acid (MG) medium (1% mannitol, 0.2% glutamic acid, 0.05% potassium phosphate monobasic, 0.02% NaCl and 0.02% MgSO_4_), *pel*-inducing minimal medium (*pel*-MM) (0.5% of polyglacturonic acid, 0.1% of yeast extract, 0.1% of (NH_4_)_2_SO_4_, 1 mM MgSO_4_ and 0.5% glycerol) or *hrp*-inducing minimal medium (*hrp*-MM) at 28°C [43–45]. *Escherichia coli* strains were cultured in LB medium at 37°C. Antibiotics were supplemented to the media at the following concentrations as needed: ampicillin (100 μg ml^-1^), kanamycin (50 μg ml^-1^) and spectinomycin (100 μg ml^-1^).

### Inoculation of *D*. *dadantii* on potato

For inoculations on potato tubers, mature tubers (*Solanum tuberosum* ‘Russet Burbank’) were dissected from the middle, and half of a tuber was placed in a 500 ml glass beaker with the dissection facing up. Three milliliters of water was added to the bottom of the beaker. Two hundred microliters of bacterial suspension (1 × 10^8^ CFU ml^-1^) in 0.5 × phosphate saline buffer (PBS) was added on top of the tuber. Beakers containing inoculated tubers were sealed with plastic wrap and incubated at 28°C. For samples with freestanding water, 500 μl of sterile distilled water was added on top of the tuber every 4 h during the incubation. Tissues from the inoculated potato tubers were collected at different time points and were used directly for microscopy, staining, or cell separation procedures. Each experiment consisted of 3–5 biological replicates, and experiments were repeated at least three times. Leaf and stem inoculations were performed using a previously described method [46].

### Microscopy

For scanning electron microscopy (SEM), potato tuber tissues with typical soft rot symptoms were collected and fixed in paraformaldehyde/ glutaraldehyde (2.5% of each compound in 0.1 M sodium cacodylate buffer) (Electron Microscopy Sciences, Hatfield, PA) at 25°C overnight. Fixed tissues were dehydrated in 25, 50, 75, and 90% ethanol for 1 hour each and in 100% ethanol three times for 30 minutes. Dehydrated samples were air dried at room temperature and mounted on aluminum mounting stubs. Images were captured using a Zeiss Sigma VP FESEM (Carl Zeiss Inc., Oberkochen, Germany).

For epifluorescence microscopy, symptomatic tissues were mounted on glass microscope slides with 1–2 μl of distilled water and covered with a coverslip. If it was necessary for cells to be fixed onto the slides to produce a still image, slides coated with agarose (5 μl of melted agarose on a glass slide to air dry) were used. Images were observed using a Zeiss Axioplan 2 fluorescence microscope (Carl Zeiss Inc) outfitted with a Zeiss AxioCam digital camera. For bacterial cell length measurement, ImageJ software [47] was used to measure at least 1,000 individual cells from at least 5 different microscopic views for each sample.

For confocal microscopy, samples were prepared using the same way as the epifluorescence microscopy. Observation was made using a Leica SP5 confocal microscope (Leica, Wetzlar, Germany), equipped with four lasers, 405 nm, multi-line Argon, 561 nm and 633 nm, and two HyD detectors.

### Separation of filamentous cells and short cells

Potato tuber was inoculated with *D*. *dadantii* in the absence of freestanding water for 48 h. Infected potato tissues were collected from P2 position and was re-suspended in a RNA Protect (Qiagen, Hilden, Germany)-water solution (ratio 1:2). The tubes were vortexed for 30 s to break up potato tissues and the intertwined bacterial cells. The resulting suspension was filtered through a 70-μm laboratory sifting mesh (Carolina Biological Supply Company, Burlington, NC, U.S.A.) to remove large plant debris. The flow-through containing bacterial cells was loaded on top of a prepared sucrose gradient solution (20%, 30%, 40%, 45% and 50%) and was centrifuged at 1,000 × g for 30 min. After centrifugation, short cells were collected from the 20% and 30% sucrose layers while the mixture cells containing enriched filamentous cells were collected from 45% and 50% sucrose layers. The enriched filamentous cells were further purified by repeating this sucrose gradient centrifugation process. Filamentous cells collected from the 45% and 50% sucrose layers of the second round purification and the short cells from both the first and second rounds of purification were used for RNA extraction and Western blot. Cell length prior to and after the sucrose gradient centrifugation procedure was visualized by microscopy. This procedure was illustrated in S2 Fig.

### Target gene-hemagglutinin (HA) tag construction and Western blot analysis

To generate the *relA*-HA and *spoT*-HA in *D*. *dadantii*, we used double crossover mutagenesis to insert a HA tag in frame at the 3’ end of the open reading frame (ORF) of *relA* and *spoT* right before the stop codon in the *D*. *dadantii* chromosome. The 3’ region of each gene ORF without the terminator was PCR-amplified with an HA Tag and terminator, and the fragment downstream the gene ORF were also amplified. The kanamycin cassette was amplified from pKD4 [48], and was cloned between two flanking regions using three-way cross-over PCR. The PCR construct was inserted into the suicide plasmid pWM91, and the resulting plasmid (pWM-relA-HA or pWM-spoT-HA) was transformed into *D*. *dadantii* 3937 by conjugation using *E*. *coli* strain S17-1 λ-pir. To select strains with HA tag, the conjugated bacteria were first grown on MG medium amended with kanamycin, and then plated on MG plates amended with kanamycin and 5% sucrose. Sequences of target genes linked with HA tag were confirmed by Sanger sequencing.

*D*. *dadantii* cells with respective HA tags were separated by sucrose gradient centrifugation and suspended in 0.5 × PBS buffer. Cells were lysed by sonication. Proteins in crude lysates were boiled and loaded onto a 10% SDS/PAGE gel. Proteins were then transferred onto a polyvinylidene fluoride membrane (Millipore, Burlington, MA, USA). Blots were washed with PBS containing 0.05% Tween-20 and probed with an anti-HA antibody (Thermo Fisher Scientific, Waltham, MA, USA). The GAPDH protein stained with Coomassie blue was used as a loading control. The blots were incubated for 5 min in enhanced chemiluminescence reagent (Bio-Rad, Hercules, CA, USA) and detected using Kodak X-ray film (Kodak, Rochester, NY, USA).

### qRT-PCR analysis

Total RNA collected from symptomatic potato tuber tissue was isolated using an RNeasy Plant Mini Kit (Qiagen). Total RNA harvested from *pel*-MM or *hrp*-MM was isolated using an RNeasy mini kit (Qiagen). Extracted RNA was treated with Turbo DNase I (Ambion, Austin, TX, USA) and cDNA was synthesized from 1 μg of treated total RNA with iScript cDNA synthesis kit (Bio-Rad, Hercules, CA, USA). The complementary cDNA level of target genes was quantified by qRT-PCR using a SsoAdvanced universal SYBR Green supermix (Bio-Rad), as described previously [46]. Data were analyzed using a relative expression software tool [49]. The expression level of *rplU* was used as an endogenous control. The experiment was repeated twice with similar results.

### RNA-Seq analysis

Total RNA quality and integrity was determined by a nanodrop (Thermo Fisher Scientific) and by an Agilent Bioanalyzer (Agilent Technologies, Santa Clara, CA, USA) respectively. RNA transcripts were enriched by selectively depleting ribosomal RNA molecules using the Ribo-Zero Bacteria Kit (Epicentre, Madison, WI, USA) and then sheared by incubation at 94°C. Following first-strand synthesis with random primers, second strand synthesis was performed with dUTP for generating strand-specific sequencing libraries. The cDNA library was then end-repaired, and A-tailed, adapters are ligated and second-strand digestion was performed by Uricil-DNA-Glycosylase. Sample concentrations were normalized to 10 nM and loaded onto Illumina Rapid or High-output flow cells at a concentration that yields 150–250 million passing filter clusters per lane. Samples were sequenced using paired-end sequencing on an Illumina HiSeq 2500 according to Illumina protocols.

*D*. *dadantii* 3937 genome (NC_014500.1) from NCBI was indexed using HISAT2 [50] and was used as the reference genome in the RNA-seq analysis. The reads obtained from the machine were trimmed for quality and length using customs scripts. The trimmed reads were then aligned with the indexed *D*. *dadantii* genome using HiSAT2. Annotation for the reference genome was obtained from the NCBI and used in the analysis. The transcript abundances were estimated using ballgown [51]. The differential gene expression was calculated using DESeq2 [52], and the results were visualized using R.

### Mutant construction and complementation

The *relA* and *spoT* single and double deletion mutations were generated by marker exchange mutagenesis. Briefly, two fragments flanking each target gene, as well as a kanamycin cassette were PCR amplified. The three fragments were linked together using a three-way cross-over PCR with kanamycin cassette flanked by the upstream and downstream sequences of the target gene. The fusion PCR was inserted into a suicide plasmid pWM91 [5]. The resulting plasmid (pWM-relA or pWM-spoT) was transformed into *D*. *dadantii* 3937 by conjugation using *E*. *coli* strain S17-1 λ-pir. To select strains with chromosomal deletions, recombinants, grown on MG medium amended with kanamycin, were plated on MG agar plates with 5% sucrose. To generate the Δ*relA*Δ*spoT* double deletion mutant, pWM-*spoT* was transformed into a *relA* marker-less mutant via conjugation. The marker-less Δ*relA* was generated by transferring plasmid pFLP-1 [5] into the Δ*relA* strain through electroporation. Transformants were plated on LB plates containing ampicillin. Colonies that lost the kanamycin resistance were selected. pFLP-1 was cured from the mutants by replica plating on MG sucrose plates. To generate complementation strains, open reading frame regions of *relA* and *spoT* were PCR amplified and cloned into a low copy number plasmid pCL1920. Marker-less deletions were confirmed by PCR. All mutants and complementation strains were confirmed by sequencing.

### ppGpp quantification by UPLC/MS/MS

Bacterial strains were grown in 500 ml LB to OD600 = 1 (10^9^ CFU ml^-1^). Cell pellet was harvested and immediately suspended in 2 ml of 100% cold methanol, vortexed, frozen in liquid nitrogen and thawed on ice. Cell suspensions were then centrifuged at 4,000 rpm for 10 min at 4°C. The bacterial supernatants were collected and stored on ice. Same extraction procedure was repeated using the remaining pellet. Methanol extracts from the two extractions were combined, freeze-dried and re-suspended in 200 μl distilled water for LC/MS/MS analysis. Pure ppGpp (TriLink Biotech, CA, USA) was used as a positive control. Mass spectrometric quantification was performed using a XEVO_TQS (Waters Corporation, MA, USA) using ultra performance liquid chromatography and tandem mass spectrometry (UPLC-MS/MS). Samples were run through an Acquity UPLC BEH C18 1.7μm column (Waters Corporation, MA, USA) maintained at 40°C. Samples were processed under the electrospray -ve (ES-) ion mode with a capillary voltage of 3 kV and a desolvation temperature of 400°C with a sample injection volume of 10 μL. Methanol (A) and 8mM DMHA+2.8nM acetic acid (B) in water were used as the two solvents under a flow rate of 0.3 ml/min. The linear gradient for solvent B was as follows: 0–10 mins, 60%; 10–15 mins, 100%. MassLynx was used as the main interface software (Waters Corporation, MA, USA). Area of the corresponding peak in each sample was quantified using ImageJ.

### Pathogenicity assay, hypersensitive response assay, and pectate lyase production assay

Pathogenicity assay was performed on potato tubers. Briefly, overnight bacterial cultures were harvested and re-suspended in 0.5x PBS to a final concentration of 1×10^8^ CFU ml^-1^. Potato tubers were dissected from the middle and a half potato was placed in a beaker with the dissected section facing up. Two hundred microliters of bacterial suspension was added on top of dissected tuber. Inoculated potato tubers were kept at 28°C with 100% relative humidity for 18 h. Weight of decayed tuber tissue was measured using an analytical balance. Hypersensitive response assay was conducted using four week old tobacco (*Nicotiana tabacum L*. cv. Xanthi) as previously described [53]. Briefly, overnight bacterial cultures were harvested and re-suspended in 0.5x PBS to a final concentration of 1×10^8^ CFU ml^-1^, and were infiltrated into leaves using needle-less syringe. Infiltrated plants were maintained in greenhouse, and HR symptoms were recorded at 20 hpi.

Plate assay for Pel production was performed as described by Matsumoto et al. [54]. Briefly, *D*. *dadantii* strains were cultured in *pel-*MM [55]. Twenty microliters of bacterial supernatant was acquired by centrifuging 1 ml of the overnight bacterial culture, and was applied to each well made in the Pel plate (1% of PGA, 1% of yeast extract, 50 μM CaCl_2_, 50 mM Tris-HCl pH 8.5, 0.8% agarose and 0.2% sodium azide) with a No. 2 cork borer (ɸ5 mm). The bottom of each well was sealed with 0.8% of molten agarose. The plates were incubated at 28°C for 20 h, and Pel production indicated by halo rings was developed by incubating the plates with 5 N of H_2_SO_4_ for 5 min.

### Motility assay

For quantification of bacterial motility from decayed potato, potato tissues inoculated with *D*. *dadantii* (*pnptII-gfp*) at 48 hpi were collected and resuspended in sterile distilled water. Cells were viewed and imaged at 400× magnification on a fluorescence microscope. Videos were captured for at least 30 seconds using screen capture tool CamStudio (https://camstudio.org/). The speed of bacterial swimming was calculated by measuring the mobile distance between two time points by using Adobe Photoshop (Adobe, San Jose, CA, USA). At least ten cells of each sample were used for speed calculation.

For swimming and swarming motility plate assays, cells were cultured in LB broth overnight, harvested and adjusted to a final concentration of 1×10^8^ CFU ml^-1^ in 0.5×PBS. Bacterial suspension of 10 μl was spotted onto the center of swimming plates (0.2% MG agar) and swarming plates (0.4% LB agar). The plates were incubated at 28°C for 19 h, and the diameter of the radial growth was measured [56]. Each experiment consisted of three biological replicates and was been repeated at least two times.

### Bacterial tolerance to oxidative stress and antibiotics

For oxidative stress assay, overnight bacterial cultures were inoculated 1:100 into fresh LB broth. After 18 h, H_2_O_2_ were added to the bacterial suspension to reach a final concentration of 10 mM. The cell suspensions were incubated for an additional 2 h at 28°C. The number of colony forming unites (CFU) was determined by serial dilution and plating at time 0 h and then every 30 min over a 2-h incubation period.

For ampicillin persistence assay, the overnight bacterial cultures were inoculated 1:100 into fresh LB broth. After 18 h, the cell suspensions were serial-diluted and plated onto LB plates with and without ampicillin (1 μg ml^-1^ or none) to determine the number of CFU.

### Progeny quantification on potato tuber

The wild type, Δ*spoT* mutant, and complementation strain of *D*. *dadantii* (with nptII-gfp) were cultured in LB broth and adjusted to the same concentration (10^8^ CFU ml^-1^). Two hundred microliters of cell suspension were added on top of dissected potato tuber, respectively. Forty-eight hours post inoculation, 30 mg of macerated potato tuber tissue was collected from the surface center of infected potato tuber respectively. The tissues were re-suspended and incubated in 10 ml of sterile distilled H_2_O in a 100 ml grass flask with shaking for 1 h at 28°C. After 1 h incubation, the bacterial suspension was serial-diluted and plated on LB Amp plates to determine the number of progeny.

### Time-lapse microscopy

Bacteria were inoculated onto a potato tuber slide in the absence of freestanding water to induce filamentous cell formation for 24 hrs. At the end of the incubation, 2 ml of sterile distilled H_2_O was added onto the tuber surface to induce the filamentous cell division. Twenty four hours later, the remaining filamentous cells collected from the surface center of the infected tuber slide was seeded and grown on the agarose pads on microscope slides and the cell division was documented using time-lapse microscopy with the protocol described by Young et al. [57].

### DAPI staining

DAPI staining was performed as described by Markus et al [58]. Briefly, a mixture of filamentous cells and short cells was collected from the surface center of the infected potato tuber, resuspended in 0.1 ml PBS buffer, and then fixed in 75% ethanol for 10 min. The fixed cell sample was collected by centrifugation and re-suspended in 20 μl of 10 mM Tris-MgCl_2_ buffer containing 1 μg ml^-1^ DAPI (10 mg ml^-1^stock), and was incubated for 15 min at 23°C. Stained cells were then imaged using excitation/emission filters at 360 nm (BW 40 nm) using a Zeiss Axio Scope (Carl Zeiss Inc) outfitted with a SPOT RT3 digital camera.

### Statistic analysis

Means and standard deviations of experimental results were calculated using Excel (Microsoft, Redmond, WA), and statistical analyses were performed using the one-way analysis of variance (ANOVA) model in the ‘stats’ package in R unless specified. All experiment included at least three biological replicates and was repeated at least three times with similar results observed.

## Supporting information

S1 Fig Microscopy observation of *D*. *dadantii* with and without *gfp* reporter. Microscopy observations of *D*. *dadantii* (upper) and *D*. *dadantii* carrying pAT-PnptII-gfp plasmid (below) during the infection of potato tuber. Samples were collected from the surface center (P2) of the infected tubers at 12 h, 24 h and 48 h.
(EPS)

S2 Fig Illustration of the separation of short cells from filamentous cells of *D*. *dadantii* by sucrose gradient centrifugation. (TIF)

S3 Fig Proportion of filamentous cells and short cells of *D*. *dadantii* upon treatment of external ppGpp. One hundred microliters of *D*. *dadantii* (10^8^ CFU ml^-1^) was inoculated onto potato tubers. Twenty microliters of ppGpp at two concentrations (5 μmol and 50μmol) were added on top of potato tubers immediately after the inoculation of the pathogen. Tubers were kept at 28°C in the absence of freestanding water for 48 hrs before cell length measurement using microscope. Error bars indicate standard errors of the means. Presence of different letters indicates significant difference (*P* < 0.05).
(EPS)

S4 Fig Swarming motility of wild type *D*. *dadantii* and ppGpp biosynthesis mutants and complementation strains. (EPS)

S1 Video Motility of filamentous cells and short cells. A mixture of filamentous cells and short cells was collected from decayed potato tuber at 24 hpi, resuspended in sterile distilled water and observed under fluorescence microscope.
(MOV)

S2 Video Time-lapse microscopy observation of division of filamentous cells of *D*. *dadantii* upon exposure to freestanding water. (MP4)

S1 Table Strains, plasmids and primers used in this study. (DOCX)
