# Supplementary figures and images for: Cell-length heterogeneity: a population-level solution to growth/virulence trade-offs in the plant pathogen Dickeya dadantii
Source: PLoS Pathog. 2019 Aug 5;15(8):e1007703. doi: 10.1371/journal.ppat.1007703 (PMC6695200; doi:10.1371/journal.ppat.1007703)

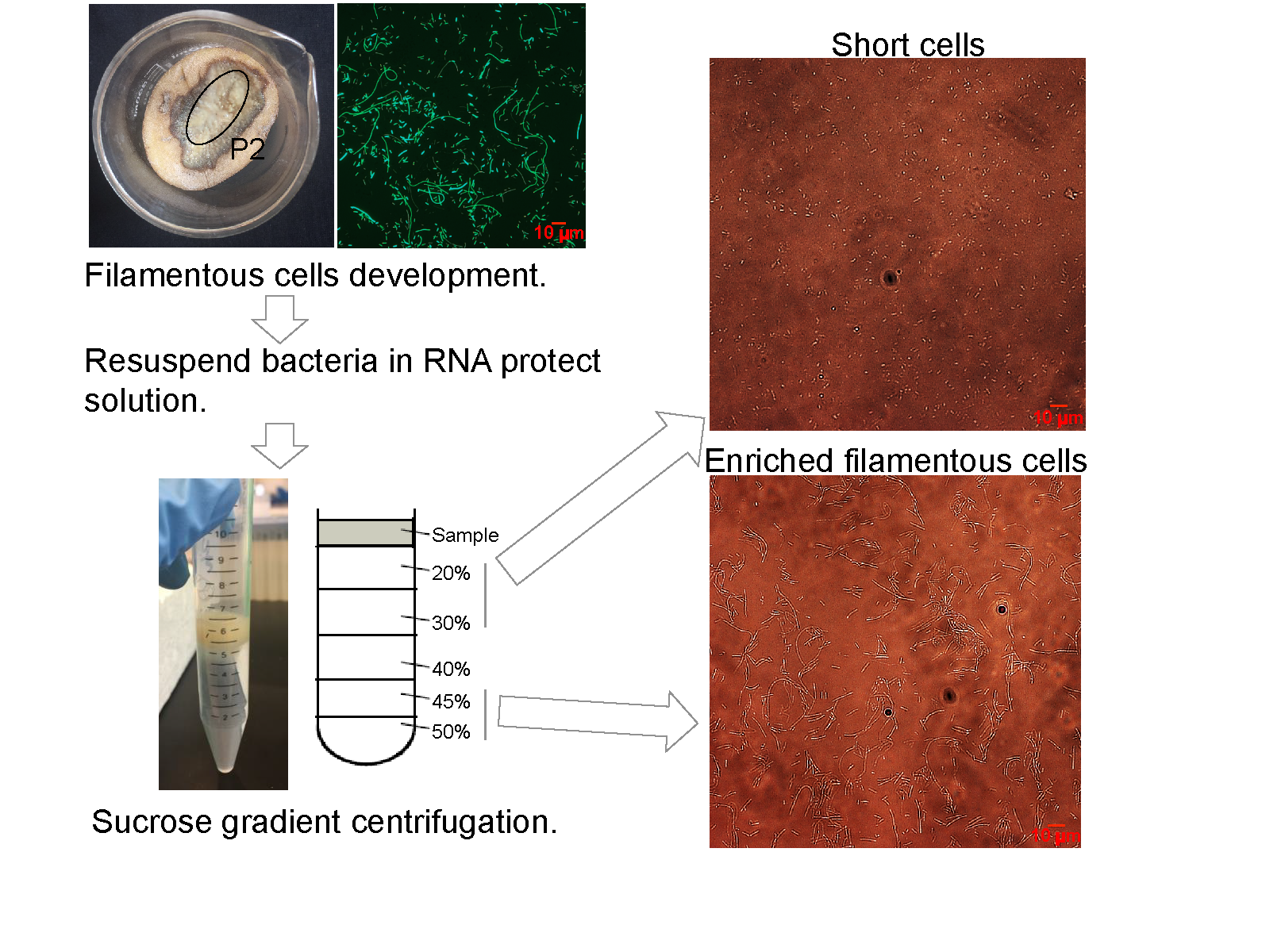

Supplement: S2 Fig — (TIF) [file ppat.1007703.s002.tif]
