# Supplementary material for: Cell-length heterogeneity: a population-level solution to growth/virulence trade-offs in the plant pathogen Dickeya dadantii
Source: PLoS Pathog. 2019 Aug 5;15(8):e1007703. doi: 10.1371/journal.ppat.1007703 (PMC6695200; doi:10.1371/journal.ppat.1007703)
Supplement: S1 Table — (DOCX) [file ppat.1007703.s007.docx]

**S1 Table. Strains, plasmids and primers used in this study.**

| **Strains, plasmids and primers** | **Characters or sequences (5' to 3') ^a^** | **Reference, source and use** |
| --- | --- | --- |
| ***Dickeya dadantii*** |  |  |
| 3937 | Wild type, isolated from *Saintpaulia* (African violet) | [1] |
| ∆*relA* | Δ*relA::Km*; Km^r^, ABF-0014861 deletion mutant | This study |
| ∆*spoT* | Δ*spoT::Km*; Km^r^, ABF-0016324 deletion mutant | This study |
| ∆*relA* ∆*spoT* | Δ*relA*Δ*spoT::Km*; Km^r^, ABF-0014861 and ABF-0016324 deletion mutant | This study |
| ∆*relA* (pCL-*relA*) | ∆*relA* containing pCL-*relA*; Sp^r^ | This study |
| ∆*spoT* (pCL-*spoT*) | ∆*spoT* containing pCL-*spoT*; Sp^r^ | This study |
| RelA-HA | *relA::HA (*before *relA* termination codon)::*Km*, Km^r^ in wide type | This study |
| SpoT*-*HA | *spoT::HA (*before *spoT* termination codon)::*Km*, Km^r^ in wide type | This study |
| ***Escherichia coil*** |  |  |
| DH5α | *supE*44 ∆*lac*U169 (*φ*80*lacZ*∆M15) *hsdR*17 *recA*1 *endA*1 *gyrA*96 *thi*-1 *relA*1 | Lab stock |
| S17-1 λpir | λ(pir) *hsdR* pro *thi*; chromosomally integrated RP4-2 Tc::Mu Km::Tn7 | Lab stock |
| **Plasmids** |  |  |
| pKD3 | Template plasmid for kanamycin cassette, Km^r^ | [2] |
| pWM91 | Sucrose-based counter-selectable plasmid, Ap^r^ | [3] |
| pFLP-2 | Suicide vector encoding flp recombinase, Ap^r^ |  |
| pCL1920 | Low copy number plasmid, lac promoter, Sp^r^ | [4] |
| pCL-*relA* | *relA* cloned in pCL1920, Sp^r^ | This study |
| pCL-*spoT* | *spoT* cloned in pCL1920, Sp^r^ | This study |
| pPROBE-AT | Promoter-probe vector, Ap^R^ | [5] |
| *nptII*-*gfp*-*hrpN*-*mCherry* | pPROBE-AT with transcriptional fusions of *nptII* promoter-*gfp* and *hrpN* promoter-*mCherry*, Ap^R^ | [6] |
| *nptII*-*gfp*-*rsmB*-*mCherry* | pPROBE-AT with transcriptional fusions of *nptII* promoter-*gfp* and *rsmB* promoter-*mCherry*, Ap^R^ | This study |
| **Primers** |  |  |
| *relA*-A-XhoI | AATACTCGAGGCGGGATTTTATTCAGGTCA | *relA* deletion |
| *relA*-B | GAAGCAGCTCCAGCCTACACCAATCTCTCCCTACTTTACCGAGC |  |
| *relA*-C | CTAAGGAGGATATTCATATGTACTGTTTTAGTACCGTAAGGCG |  |
| *relA*-D-NotI | AATATTATGCGGCCGCCCTTGGTCTGCTCCCAGTTA |  |
| *spoT*-A-Xhol | AATACTCGAGGGCGGAAATGAGTCACTACGCC | *spoT* deletion |
| *spoT*-B | GAAGCAGCTCCAGCCTACACCGGGCGACTCGCGACCGAA |  |
| *spoT*-C | CTAAGGAGGATATTCATATGCCTGTTATGACCCCAGAACGT |  |
| *spoT*-D-NotI | AATATTATGCGGCCGCGCTGCTGTTCTTCCTCATCC |  |
| Km-F | TGTGTAGGCTGGAGCTGCTTCG | Km cassette |
| Km-R | CATATGAATATCCTCCTTAGTTCCTATTCC |  |
| *relA*­-for-XbaI | TGCTCTAGAATGGTTGCGGTAAGAAGTGC | *relA* complementation |
| *relA-*rev-SacI | CGAGCTCTATCCCACCAGGCGTCAT |  |
| *spoT*-for-XbaI | TGCTCTAGATTGTATCTGTTTGAAAGCCTCA | *spoT* complementation |
| *spoT-rev-SacI* | CGAGCTCTTAATTTCGGTTACGGTTGACTT |  |
| *relA* HA-A-XhoI | AATACTCGAGGCCAACTGACGCAGAAATC | *relA* HA |
| *relA* HA-B | GAAGCAGCTCCAGCCTACACCTCAAGCGTAATCTGGAACATCGTATGGGTAATTGCCCTGCAACCGC |  |
| *relA* HA-C | CTAAGGAGGATATTCATATGTACTCCACATCATGCAGCG |  |
| *relA* HA-D-NotI | AATATTATGCGGCCGCACCATCACCTCGTCGATCTC |  |
| *spoT* HA-A-XhoI | AATACTCGAGGGTAACGCTATGAGCGTGGT | *spoT* HA |
| *spoT* HA-B | GAAGCAGCTCCAGCCTACACCTTAAGCGTAATCTGGAACATCGTATGGGTAATTTCGGTTACGGTTGACTTT |  |
| *spoT* HA-C | CTAAGGAGGATATTCATATGCCTGTTATGACCCCAGAACG |  |
| *spoT* HA-D-NotI | AATATTATGCGGCCGCTGCGCTTCGTACAGAATCAA |  |
| *pelD*-F | GAGTGGGATGCGGCGGTGAT | *pelD* qPCR |
| *pelD*-R | AGCGGCTGCTGGAAATGG |  |
| *pelE*-F | GAAAGCCCGCAGCCAGAT | *pelE* qPCR |
| *pelE*-R | CGGCGTTCCAACCATCTC |  |
| *hrpA*-F | CAGCAATGGCAGGCATGCAG | *hrpA* qPCR |
| *hrpA*-R | CTGGCCGTCGGTGATTGAGC |  |
| *hrpN*-F | TCGGCAGCGGTCTGAACGAC | *hrpN* qPCR |
| *hrpN*-R | CCAGCGACAACGGCGAGAA |  |
| *dspE*-F | GATGGCGGAGCTGAAATCGTTC | *dspE* qPCR |
| *dspE*-R | CCTTGCCGGACCGCTTATCATT |  |
| *hrpL*-F | GATGATGCTGCTGGATGCCGATGT | *hrpL* qPCR |
| *hrpL-R* | TGCATCAACAGCCTGGCGGAGATA |  |
| *relA-*F | CTGCTGCCGCGTTACAACAT | *relA* qPCR |
| *relA-*R | CCGGTTGCTGGGATTTCTGC |  |
| *spoT-*F | ATCATGGCGATGGTGCAGGA | *spoT* qPCR |
| *spoT-*R | CCCAGCTCTTCCAGTTCGGT |  |
| *gyrA*-F | TGATGGAAGTGATCCGTGAA | *gyrA* qPCR |
| *gyrA*-R | GGTCACCACCACGTTCTCTT |  |
| *rplU*-F | GCGGCAAAATCAAGGCTGAAGTCG | *rplU* qPCR |
| *rplU*-R | CGGTGGCCAGCCTGCTTACGGTAG |  |

^a^ Ap^R^ indicated ampicillin resistance; Km^r^, kanamycin resistance; Sp^r^, spectinomycin resistance; sequence underline indicated restriction enzyme sites; *gfp*: green fluorescent protein gene, *mCherry*: red fluorescent reporter protein gene.

1. Yang C-H, Gavilanes-Ruiz M, Okinaka Y, Vedel R, Berthuy I, Boccara M, Chen JW-T, Perna NT, Keen NT: **hrp genes of Erwinia chrysanthemi 3937 are important virulence factors**. *Molecular plant-microbe interactions* 2002, **15**(5):472-480.

2. Datsenko KA, Wanner BL: **One-step inactivation of chromosomal genes in Escherichia coli K-12 using PCR products**. *Proceedings of the National Academy of Sciences* 2000, **97**(12):6640-6645.

3. Metcalf WW, Jiang W, Daniels LL, Kim S-K, Haldimann A, Wanner BL: **Conditionally Replicative and Conjugative Plasmids Carrying *lacZ* α for Cloning, Mutagenesis, and Allele Replacement in Bacteria**. *Plasmid* 1996, **35**(1):1-13.

4. Lerner CG, Inouye M: **Low copy number plasmids for regulated low-level expression of cloned genes in *Escherichia coli* with blue/white insert screening capability**. *Nucleic Acids Research* 1990, **18**(15):4631.

5. Miller WG, Leveau JH, Lindow SE: **Improved gfp and inaZ broad-host-range promoter-probe vectors**. *Mol Plant Microbe Interact* 2000, **13**(11):1243-1250.

6. Zeng Q, Laiosa MD, Steeber DA, Biddle EM, Peng Q, Yang C-H: **Cell individuality: the bistable gene expression of the type III secretion system in Dickeya dadantii 3937**. *Molecular plant-microbe interactions* 2012, **25**(1):37-47.
